# The OPtimising HEalth LIterAcy (Ophelia) process: study protocol for using health literacy profiling and community engagement to create and implement health reform

**DOI:** 10.1186/1471-2458-14-694

**Published:** 2014-07-07

**Authors:** Roy W Batterham, Rachelle Buchbinder, Alison Beauchamp, Sarity Dodson, Gerald R Elsworth, Richard H Osborne

**Affiliations:** 1 Public Health Innovation, Population Health Strategic Research Centre, School of Health and Social Development, Deakin University, 221 Burwood Highway, Melbourne, Victoria 3125, Australia; 2 Monash Department of Clinical Epidemiology, Cabrini Hospital, Malvern VIC, Australia; 3 Department of Epidemiology and Preventive Medicine, School of Public Health and Preventive Medicine, Monash University, Melbourne, Australia

**Keywords:** Health literacy, Equity, Chronic illness, Access, Implementation, Intervention development, Intervention mapping, Participatory research, Health Literacy Questionnaire (HLQ), Co-creation

## Abstract

**Background:**

Health literacy is a multi-dimensional concept comprising a range of cognitive, affective, social, and personal skills and attributes. This paper describes the research and development protocol for a large communities-based collaborative project in Victoria, Australia that aims to identify and respond to health literacy issues for people with chronic conditions. The project, called Ophelia (OPtimising HEalth LIterAcy) Victoria, is a partnership between two universities, eight service organisations and the Victorian Government. Based on the identified issues, it will develop and pilot health literacy interventions across eight disparate health services to inform the creation of a health literacy response framework to improve health outcomes and reduce health inequalities.

**Methods/Design:**

The protocol draws on many inputs including the experience of the partners in previous co-creation and roll-out of large-scale health-promotion initiatives. Three key conceptual models/discourses inform the protocol: intervention mapping; quality improvement collaboratives, and realist synthesis. The protocol is outcomes-oriented and focuses on two key questions: ‘What are the health literacy strengths and weaknesses of clients of participating sites?’, and ‘How do sites interpret and respond to these in order to achieve positive health and equity outcomes for their clients?’. The process has six steps in three main phases. The first phase is a needs assessment that uses the Health Literacy Questionnaire (HLQ), a multi-dimensional measure of health literacy, to identify common health literacy needs among clients. The second phase involves front-line staff and management within each service organisation in co-creating intervention plans to strategically respond to the identified local needs. The third phase will trial the interventions within each site to determine if the site can improve identified limitations to service access and/or health outcomes.

**Discussion:**

There have been few attempts to assist agencies to identify, and respond, in a planned way, to the varied health literacy needs of their clients. This project will assess the potential for targeted, locally-developed health literacy interventions to improve access, equity and outcomes.

## Background

The World Health Organisation describes health literacy as “the cognitive and social skills which determine the motivation and ability of individuals to gain access to, understand and use information in ways which promote and maintain good health” [1]. While the competencies of individuals and other social decision-making units (e.g., families) are central to the concept, increasingly government, health and community services, consumer groups and researchers are recognising their responsibilities to respond appropriately and effectively to the health literacy needs of the consumers they serve and represent [2-6].

To date, measures of health literacy have focused on a limited range of health-related literacy and numeracy skills [7]. Many studies have demonstrated associations between these measures and health and health-related outcomes. These include increased hospital admissions and readmissions [8-12]; less participation in preventive activities [12-16]; higher prevalence of health risk factors [17,18]; poorer self-management of chronic conditions [18-22] and poorer disease outcomes [22,23]; lower functional status [24]; and increased mortality [25-27]. A 2009 review estimated that limited reading- and numeracy-related health literacy accounted for an additional 3-5% of total healthcare cost annually, or up to an additional US$7,798 per year for individual patients [28]. Studies also suggest that differences in health literacy abilities may explain observed health inequalities among people of different race and with differing levels of educational attainment [29,30]. Developing interventions to address low health literacy therefore provides an opportunity to improve health outcomes and reduce health inequalities.

There are many challenges associated with the development of effective responses to health literacy needs, and these highlight the importance of a systematic development approach [31]. We have demonstrated that health literacy is a multi-dimensional concept that contains a variety of cognitive, affective, social, and personal skills and attributes [32,33]. This suggests the potential for a diversity of needs and strengths in the health literacy of individuals and groups, both over time and across the dimensions of health literacy. It also suggests that interventions designed and tested in one setting or with one population may have limited applicability in other settings and populations.

Fortunately, recent developments in the measurement of health literacy have increased capacity to assess needs across a more extensive set of dimensions of health literacy. The Health Literacy Questionnaire (HLQ), for example, provides detailed insights into health literacy needs and strengths across nine distinct domains [34]. This advance in measurement creates the potential for providers, organisations and governments to identify and understand the health literacy ‘profiles’ of individuals and/or populations. For the first time, use of such data allows development and selection of ‘fit-for-purpose’ health literacy responses that are comprehensive, and optimise opportunities to improve access, equity and outcomes.

Given that the capacity to measure and diagnose needs and strengths across distinct health literacy domains is new, questions remain regarding *what* strategies are appropriate and effective, for *which* organisations and individuals. Building an evidence base that responds to these questions is a long-term endeavour. The protocol described here, however, outlines how the Ophelia Victoria study is seeking to make a start in this field. This study proposes the development of response ideas and evidence relating to endemic problem areas, such as poor access to services by the most high-risk and disadvantaged groups; low rates of participation in preventive services; and difficulties in achieving or maintaining behaviour change.

Specifically, this paper outlines the protocol for the development and testing of health literacy interventions across eight health and community care organisations in Victoria, Australia. We refer to the proposed process as ‘the Ophelia process’. The process is outcomes-oriented and focuses on two key questions: ‘What are the health literacy strengths and weaknesses of clients of participating sites?’, and ‘How do sites interpret and respond to these in order to achieve positive health and equity outcomes for their clients?’. The Ophelia process uses the HLQ as the foundation for identification of the health literacy needs of each organisations’ local community. The outcome of the project will be a framework that provides intervention options for organisations to employ when members of the community present with particular health literacy needs. It also seeks to offer proof of concept that tailored responses to local health literacy needs are feasible, and can help improve service access, health behaviour, and health outcomes.

## Methods

### Methodological foundations

The approach to building and refining interventions is based on three systems that have been utilised for collaborative intervention development, intervention optimisation and shared learning: intervention mapping (IM), quality improvement collaboratives (QIC), and realist synthesis.

The Ophelia process utilises an adaptation of IM that draws on some of the effective elements of the QIC method. IM was developed to guide the process of intervention development and implementation [35,36]. It involves six steps (described in detail elsewhere [35,37]). The first step of IM is a needs assessment [38] that ensures the development of a clear and comprehensive description of the health problem in question, its impacts on health and quality of life, related behavioural and environmental conditions, and any known determinants.

In practice, the Ophelia process will begin with a structured needs assessment, which will be carefully fed back to participating organisations. The organisations will then be engaged in a collaborative process of intervention identification and development. Organisations will co-create and refine interventions using Plan-Do-Study-Act (PDSA) cycles and be supported to collaborate with one another through formal communities of practice. This application of IM draws heavily from the QIC method, which has been used in a variety of settings to assist service providers to optimise quality of care, access and outcomes. It uses three interacting processes: 1) providing feedback on comparative data and assisting services to monitor their own data over time; 2) providing training in quality improvement methods such as PDSA cycles; and 3) providing opportunities to share ideas about methods for improvement (communities of practice). Systematic reviews about the utility of the QIC method are mixed [39-41]. There is increasing evidence, however, that if sufficient attention is paid to strengthening the local team [40,42], improvements in the consistency and quality of care can be demonstrated [43], as can improvements in access and outcomes [44].

Finally, the Ophelia process draws upon realist methods. Realist methods are best known in the health sector through the recent popularity of realist synthesis [45-49]. The central feature of realist methods is the focus on the mechanisms by which programs have an effect, and the recognition that different mechanisms may be activated by different interventions in different contexts and for different people [49,50]. Pawson and Tilleys’ realist approach integrates knowledge by identifying ‘context’, ‘mechanism’ and ‘outcome’ configurations (CMOs) at the level of the unit that is the focus of the intervention [47]. We have added the ‘intervention’ by which the mechanism is activated, which Pawson and Tilly merged with mechanisms. Thus, we refer to CIMOs. These local analyses are then synthesised to achieve increasingly general insights into which mechanisms are important in different situations and for different people, and how the key mechanisms can be activated successfully in different circumstances. It is the focus on contexts and mechanisms that makes it an overtly realist approach.

### Partnerships and funding

The project is funded through an Australian Research Council Linkage project grant. These grants aim to support research and development projects undertaken to acquire new knowledge, and which involve risk or innovation. Linkage projects are collaborations between higher education researchers (in this case, Deakin University and Monash University) and non-academic sectors (in this case, the Victorian Department of Health).

### Ethics

Ethics approval for the study has been obtained from the Human Research Ethics Committee of Deakin University and from four of the participating sites. Approval to conduct the study at the remaining four sites was included within the Deakin University approval process. Written informed consent will be obtained from all participants.

### Participating sites

Health and community service organisations from four of the nine health department regions of Victoria—representing an array of socio-demographic characteristics—were invited to respond to a call for ‘expressions of interest’. Criteria for the selection of sites were a) evidence of executive engagement and commitment to the project; b) sufficient staff and client numbers; c) location of the service (to ensure an appropriate mix of rural, regional, and metropolitan sites); d) presence of a management structure and organisational culture that supports service development and quality improvement activities; e) a track record in engaging and delivering on quality improvement initiatives (sought a mix of sites with high and low experience); and f) a willingness and capacity to commit in-kind resources (in particular, staff time to participate in project activities). Selected sites were located in metropolitan and rural Victoria and included two local government organisations, one outreach nursing service, two hospital-based sub-acute programs, and three community healthcare organisations.

### Materials

The HLQ will be used as the primary measure of health literacy [34]. It contains 44 questions across nine domains:

1) Feeling understood and supported by healthcare providers

2) Having sufficient information to manage my health

3) Actively managing my health

4) Social support for health

5) Appraisal of health information

6) Ability to actively engage with healthcare providers

7) Navigating the healthcare system

8) Ability to find good health information

9) Understand health information well enough to know what to do

The HLQ was designed using a validity-driven approach [51] and validated in diverse samples of individuals in the community. It has been shown to have strong construct validity, reliability and high acceptability to clients and clinicians [34]. It was designed for administration by pen and paper self-administration or by interview to ensure inclusion of people who cannot read or have other difficulties with self-administration. It is available in many languages and has been used in many studies to inform intervention development as well as in surveys and evaluations.

### Study design and participants

**Figure 1:**
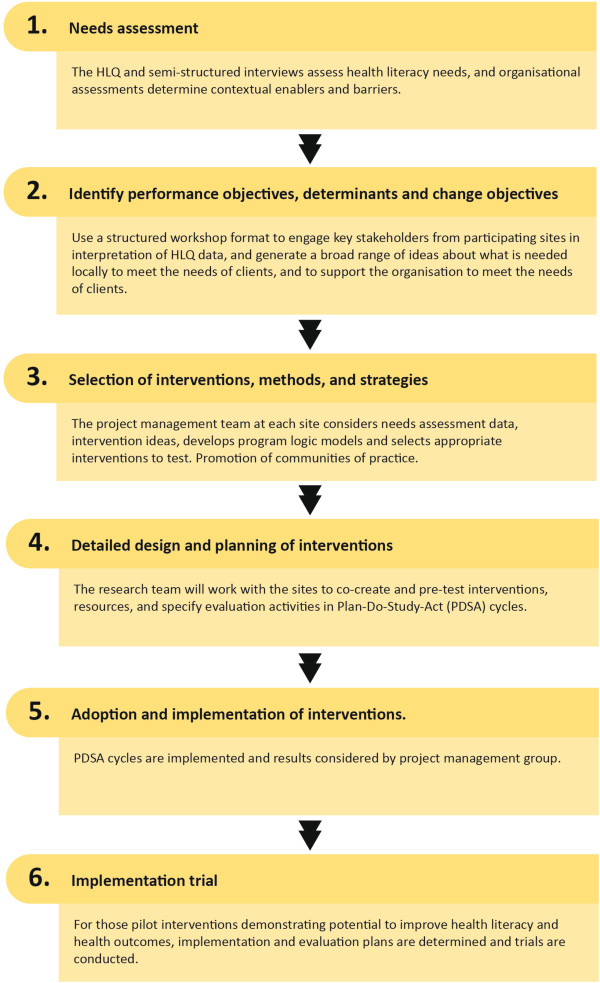
Steps in the Ophelia process for development of health literacy interventions.

A modified intervention mapping approach will be employed for this study [36]. Project phases are shown in Figure 1 and include the following six main steps:

#### Needs assessment

An assessment of health literacy needs and organisational structures across the eight participating sites will be performed. Data collection will be led and undertaken by the site project management teams.

The HLQ and demographic data will be collected from a minimum of 100 clients from each site. A mix of recruitment approaches and questionnaire administration options will be employed to ensure that the sample includes people with limited literacy, people from culturally and linguistically diverse backgrounds, people with complex medical or psychosocial needs, and people from other disadvantaged groups. Due to the difficulties associated with administering surveys to people with low health literacy and stratifying in advance, it is not expected that the survey data will fully represent the population of the health service. Organisations will, however, be asked to fulfil sampling quotas of people who they estimate to have high and low health literacy.

Cluster analysis of data from the HLQ will be used to identify health literacy profiles of groups of individuals within each sample. Semi-structured interviews will also be conducted with 10 to 12 consumers at each site, stratified by the identified clusters. These data will then be synthesised with the HLQ cluster results to generate 5 to 8 vignettes of the typical groups of clients attending the organisation across health literacy levels. These vignettes will provide a tangible description of the health literacy needs of a comprehensive range of target clients. The vignettes and other HLQ and demographic data will then be presented at consultation workshops involving experienced practitioners and managers at each site. Workshop participants will be invited to consider the vignettes and identify intervention ideas that they believe would 1) enhance the health literacy of their target population; 2) improve their organisation’s response to the identified health literacy needs of their community; and 3) involve the local community in social development activities with potential to improve health literacy.

**Figure 2:**
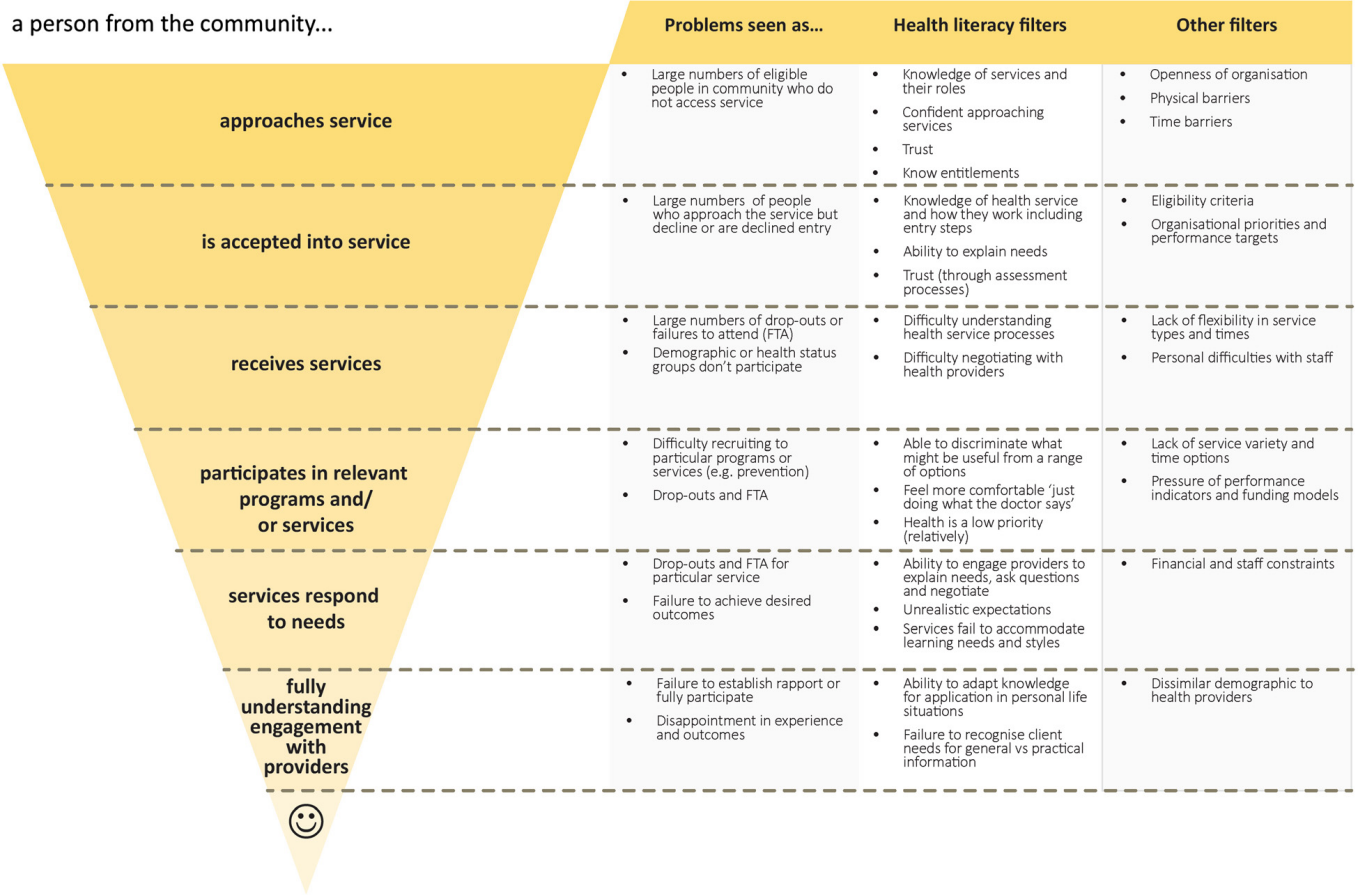
Filters that determine a person’s participation and inclusion in healthcare.

The systematic assessment of each organisation will involve a series of semi-structured interviews with service providers, consumers, managers and state government representatives. Organisations will also provide information about their strategic priorities and plans, organisational structure, the local service system environment, and socio-demographic data pertaining to the population they serve.The final stage of the needs assessment will consider how the identified health literacy issues may be affecting service access, equity and outcomes, and identify priorities for intervention. This process requires integration of data about health literacy with data from the site and will be guided by the model presented in Figure 2. Figure 2 suggests ways in which health literacy influences the extent to which people engage with and benefit from health services. At each level of the model it is likely to be people with high health risks and/or low health literacy who are filtered out and therefore do not derive full benefit from the organisations’ services. In this stage of the needs assessment, participating sites must answer four key questions:

1) Who are the clients or potential clients who are missing out the most (considering both numbers and health risk), and at what level are they being filtered out?

2) In what ways could health literacy deficits be contributing to the problem?

3) What other issues could be contributing?

4) What short-term outcome indicators could be used to monitor the effectiveness of trial interventions?

#### Identify performance objectives, determinants and change objectives

At the commencement of this phase, key stakeholders from each site will be invited to participate in planning activities. The first of these will use the needs assessment data to begin construction of site-based logic models. The first and simplest model is referred to as an ‘outcomes hierarchy’ which will detail the desired program outcomes (related to Figure 2), and how the identified health literacy needs may affect these outcomes. The logic models will then identify key intermediate outcomes and short to medium term change indicators [52-54]. (Note especially Supplementary Appendix 2 to Porter, 2010). This detail will then be used to focus the ideas generated during consultation workshops and subsequent decisions about interventions to test during pilot activities.

#### Selection of interventions, methods, and strategies

Local providers will identify interventions that they perceive have the potential to achieve the desired organisational and client outcomes. When sites have identified one or more intervention ideas from the pool of ideas generated during consultation workshops, they will draft a McClintock-style program logic which identifies the mechanisms that may produce benefit [55,56]. Once the models have been developed, external theories and evidence will be interrogated to identify conditions under which a given approach is likely to be effective and critique the assumptions of the local model.

Initial intervention ideas will be shared at a workshop involving all sites. Sites will be given the opportunity to revise their models and planned interventions based on feedback from the other sites and the research team, and/or evidence from the literature where this is available. Sites will be assisted to partner together to develop a community of practice. Data and program logic models from all sites will be synthesised to allow development of a project level program logic that will include details of the potential mechanisms by which interventions can address identified health literacy needs.

#### Detailed design and planning of interventions

This stage will involve specifying the scope and sequence of intervention activities and the required resources and materials. Training programs, decision supports, templates and guidelines will then be sourced or developed. The research team will work with the sites to co-create and pre-test these interventions, resources, and specify evaluation activities in PDSA cycles. Participants required for the intervention trial will be determined, as will determinants of adoption, implementation and sustainability.

#### Adoption and implementation of interventions

This action phase will be structured around a set of milestones identified in step 4 and it will include a series of PDSA cycles. There will be increased emphasis on the sharing of discoveries across sites through the communities of practice.

The aim of steps 4 and 5 is to ensure that the sites have implementable interventions in place for testing during the final stage.

#### Implementation trial

Immediate and intermediate health and quality of life outcomes will be examined. Important immediate outcomes will relate to a) the specific HLQ domains that were identified as problematic, and b) the access, participation and outcomes issues from Figure 2 that the organisation identified as priorities. Thus, for some organisations, outcomes may be behaviour change for people participating in preventive programs while, for others, outcomes may relate to the proportion of eligible clients that chooses to participate in preventive programs at all.

Sites will examine the health literacy outcomes associated with the interventions they test using a few relevant HLQ scales, and a website will be available to assist them to collect, analyse and interpret the results. It is anticipated that the website will also provide a venue for service providers and managers to collaborate, share ideas and resources, and to communicate key findings. The expectation is that it will be widely available to national and international healthcare organisations that wish to use the HLQ and the Ophelia process as mechanisms to drive quality improvement, and to generate innovations to improve health outcomes and reduce health inequalities.

### Data collection, management and analysis

**Figure 3:**
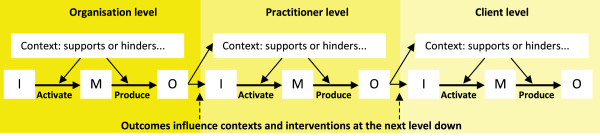
Context, interventions, mechanism and outcomes (CIMO) configurations across organisation, practitioner and client levels.

This is a complex project that needs to capture evolving ideas about strategies to respond to identified health literacy needs; strategies that may operate at the client, practitioner, organisational or inter-organisational level. There is a need to manage data from a wide variety of sources and to integrate qualitative and quantitative data sources. The data management and analysis systems must also enable the exploration of relationships between contexts, interventions, mechanisms and outcomes. In addition, it is necessary to consider a chain of possible interventions at organisation, practitioner and client levels in which the outcomes at each level feed into the context and capacities at the next level (see Figure 3). The analysis could be called a causally and hierarchically ordered meta-matrix [57]: ‘causally ordered’ in that there is an implied direction of influence from context and interventions to mechanisms to outcomes; ‘hierarchical’ in that it considers organisation, practitioner and client levels; and ‘meta-matrix’ in that the same data will be collected across different service types and related to different health literacy needs. A matrix model of this complexity usually requires computer-assisted management and will be developed in NVivo 10 software [58].Some of the data will relate to pre-defined categories and concepts, whereas other data will be collected to respond to emerging insights and issues as all participating sites work together on co-discovery, co-creation and supported problem solving. The scales of the HLQ provide one structuring element in the data in that intervention ideas will be related to low scores on the various scales (or to particular profiles of scores across the scales). The sequence of CIMOs illustrated in Figure 3 provides a broad organising framework but it is expected that considerable detail will emerge related to each box and arrow and that sophisticated qualitative coding and analysis tools will be needed to ensure that emerging issues are identified, clarified and verified with as much rigour as possible.

Data will be collected and synthesised throughout the project, and the nested logic models will be continually refined. Data will be collected at four levels:

a) Implementation indicators (specific to the focus chosen by the site)

b) Clinical management

c) Service redesign in response to health literacy needs assessment

d) Outcomes

In addition, notes on all key decision-making activities will be entered into NVivo 10 [58] and coded with an emphasis on a) identifying CIMO elements; b) tracking reasoning and emergent learning related to intervention ideas; and c) identifying responses to specific health literacy needs. The activities that will be included will comprise all workshops with sites; all research team meetings; correspondence related to refining interventions; reports on site assessments and interviews; an issues log.

The sequence of workshops with participating sites will be a key analytic and learning activity. Sites will develop increasingly sophisticated and action-oriented program logic models, and between each step the research team will be considering relevant additional inputs such as theories and evidence from the literature and/or additional data from the sites.

At the client level, intensive case studies—incorporating health literacy profiles, service utilisation and outcome data, and interviews—will be developed to elaborate CIMOs for a sample of individuals with varied health literacy needs, as indicated by their profile of scores across the HLQ scales.

### Quantitative analysis

Demographic data will be analysed using descriptive statistics. HLQ domains will be presented as a mean with 95% confidence intervals, or as median scores with interquartile ranges. Cluster analysis will be used to group data (hierarchical approach using Ward’s method for linkage). The number of clusters identified for presentation at each site will be guided by a) seeking to minimise the remaining variance within each scale within each cluster (e.g., if there are standard deviations greater than 0.6 for one or more of the scales it may indicate that there are still significant subgroups within the cluster); and b) ensuring that clusters represent different patterns of needs and strengths across the nine scales of the HLQ.Wherever possible the outcomes related to Figure 2 will use routine service data that has been available for an extended timeframe. This is assisted by the fact that services funded by the Victorian Department of Health have a standard minimum data set including intake characteristics and service delivery. This will allow for an extended baseline data set with trends over time in most instances.

### Sample size

Pre-implementation activities at each site will include a sufficient number of consumers/practitioners to demonstrate proof of concept. The content of the final interventions at each site may range from organisational policy, practitioner training, community empowerment, to specific interventions with clients. Consequently both qualitative and quantitative evaluation methods will be used and sample size decisions will be undertaken in response to the specific evaluation method and the nature of the intervention emerging at each site. For example, the intervention may include all eligible clients entering the service during a set period of time, or it may involve a sufficient number of clients to detect a medium to large effect size across specific HLQ domains (i.e., pre-post change equivalent to a change of at least 0.5 standard deviations using alpha = 0.05 and beta = 0.9).

## Discussion

Although the potential of health literacy has been recognised in academic publications [3,6] and policy documents [59-61], a mechanism by which the concept can be operationalised on a large scale, and in a way that is responsive to different health literacy needs in the population, has not been forthcoming. The proposed project will create health literacy interventions that will be developed and trialled in eight organisations across metropolitan and rural Victoria, with the aim of generating best practice across the targeted populations. The project will provide insights into health literacy research and practice and will provide a framework that can be applied in a wide range of contexts.

The proposed research directly investigates and tackles health literacy as a cause of social inequalities in health, and provides partnership and policy-driven approaches to innovation generation and system reform. If nations are to reduce the growing burden of chronic diseases and the widening health gap between rich and poor, then innovative approaches are required to empower and inform consumers, practitioners and policymakers. To this end, this project will generate new data and tools to inform practice and policy, and enable practitioners at both the patient and organisation levels to understand and meet the needs of the community, targeting those who are disadvantaged.

The proposed project is innovative in that the research a) recognises that health literacy is multidimensional and different people may have different patterns of health literacy needs; b) takes a systematic and grounded approach to intervention development; c) co-creates new health literacy interventions with stakeholders across multiple organisational levels (government, community health and local councils) to maximise applicability and penetration across the community; and d) generates tools and interventions that will have high utility and uptake because ownership is generated with future users.

While this study will not generate evidence about the effectiveness of specific health literacy interventions, its purposes are to provide a framework for intervention responses; to generate a wide range of intervention ideas based on the experience of the best practitioners as well as the literature; and to provide a proof of concept that by responding to identified health literacy needs, healthcare organisations can address identified service access and effectiveness issues and improve outcomes in relation to these issues. The project outcomes will guide both activity and knowledge integration in a much larger program of research that, it is hoped, will engage researchers around the world.

The response framework will consider several issues of context including the specific purposes and target groups of the participating organisations; socio-economic and demographic differences; and health literacy differences (which in a realist framework should be considered part of the context rather than a mechanism or outcome). There is also a larger context of a relatively affluent Australian society with a high level of public services and universal health insurance. It is expected, however, that the methods could be applied in countries and situations with very different contexts in order to build response frameworks suitable to those contexts.

It is also recognised that there will be a need in the future for more substantial trials to demonstrate a) the effectiveness of health literacy needs assessment and response planning as a means for agencies to improve outcomes in relation to identified limitations to equity and effectiveness; and b) the utility of many of the specific intervention ideas. With regards to the latter, however, the Ophelia approach to development of health literacy interventions emphasises customising care to individual client needs rather than applying a standardised intervention. Similarly at an organisational level, the Ophelia process emphasises problem solving in context rather than promoting mimicry of specific interventions.

## Conclusion

This research protocol will test a new model of health literacy intervention development and application. It will advance the understanding of health literacy and how it can be used to improve health outcomes. It will also derive new knowledge about the role of health literacy as both a determinant of, and mechanism to reduce, health inequalities.

## Abbreviations

CIMO: Context, intervention, mechanism and outcome configuration; HLQ: Health Literacy Questionnaire; IM: Intervention mapping; QIC: Quality improvement collaborative; PDSA: Plan-Do-Study-Act.

## Competing interests

The authors declare that they have no competing interests.

## Authors’ contributions

The overall study design was led by RWB, RHO and RB, which lead to the successful ARC Linkage (nationally competitive) grant application. RWB and GRE led the realist synthesis methods. AB and SD wrote the initial draft and detailed project plan. All authors contributed to redrafting and approving the final draft.

## Pre-publication history

The pre-publication history for this paper can be accessed here:

http://www.biomedcentral.com/1471-2458/14/694/prepub
